# Genetic variants within the *hTERT* gene and the risk of colorectal cancer in Lynch syndrome

**DOI:** 10.18632/genesandcancer.85

**Published:** 2015-11

**Authors:** Aung Ko Win, Mark Clendenning, William Crawford, Christophe Rosty, Susan G. Preston, Melissa C. Southey, Susan Parry, Graham G. Giles, Finlay A. Macrae, Ingrid M. Winship, John A. Baron, John L. Hopper, Mark A. Jenkins, Daniel D. Buchanan

**Affiliations:** ^1^ Centre for Epidemiology and Biostatistics, Melbourne School of Population and Global Health, The University of Melbourne, Parkville, Victoria, Australia; ^2^ Colorectal Oncogenomics Group, Genetic Epidemiology Laboratory, Department of Pathology, The University of Melbourne, Parkville, Victoria, Australia; ^3^ Envoi Specialist Pathologists, Herston, Queensland, Australia; ^4^ University of Queensland, School of Medicine, Herston, Queensland, Australia; ^5^ Genetic Epidemiology Laboratory, Department of Pathology, The University of Melbourne, Parkville, Victoria, Australia; ^6^ New Zealand Familial Gastrointestinal Cancer Service, Auckland, New Zealand; ^7^ Cancer Epidemiology Centre, Cancer Council Victoria, Melbourne, Victoria, Australia; ^8^ Department of Medicine, The University of Melbourne, Parkville, Victoria, Australia; ^9^ Genetic Medicine and Family Cancer Clinic, Royal Melbourne Hospital, Parkville, Australia; ^10^ Colorectal Medicine and Genetics, The Royal Melbourne Hospital, Parkville, Victoria, Australia; ^11^ Department of Medicine, University of North Carolina, Chapel Hill, North Carolina, USA; ^12^ Department of Epidemiology and Institute of Health and Environment, School of Public Health, Seoul National University, Seoul, Korea

**Keywords:** Lynch syndrome, hTERT, colorectal cancer, genetic modifier, genetic variant

## Abstract

Lynch syndrome is an inherited cancer-predisposing disorder caused by germline mutations in the DNA mismatch repair (MMR) genes but there is a high degree of variability in cancer risk observed among carriers, suggesting the existence of modifying factors. Our aim was to investigate variants within the *hTERT* gene as a potential colorectal cancer (CRC) risk modifier for MMR gene mutation carriers. We identified 1098 MMR gene mutation carriers (420 *MLH1*, 481 *MSH2*, 126 *MSH6*, 53 *PMS2* and 18 *EPCAM*) from 330 families recruited from either family cancer clinics or population cancer registries of the Australasian Colorectal Cancer Family Registry between 1997 and 2012. Using weighted Cox regression after adjusting for ascertainment bias, we estimated associations between 23 SNPs within the *hTERT* gene and CRC risk. During 46,836 person-years observation, 392 (36%) carriers were diagnosed with CRC at a mean age of 42.2 (standard deviation 11.4) years. There was no evidence of association between any of the *hTERT* SNPs and CRC risk, overall and stratified by sex and MMR gene mutated, after adjustment for multiple testing. Our findings suggest no evidence for clinical utility of the SNPs within the *hTERT* gene in Lynch syndrome.

## INTRODUCTION

Lynch syndrome, formerly known as hereditary non-polyposis colorectal cancer, is an autosomal dominant disorder caused by germline mutations in one of the DNA mismatch repair (MMR) genes *MLH1*, *MSH2*, *MSH6*, and *PMS2*, or *EPCAM*. MMR gene mutation carriers are at increased risk of developing cancers of the colorectum and endometrium, as well as cancers of the ovary, kidney, pancreas, stomach, and urinary bladder. The risk of colorectal cancer (CRC) to age 70 years for MMR gene mutation carriers is reported to be between 12 and 50% [1, 2]. In addition to environmental factors such as obesity and smoking [3], there is evidence suggesting the existence of genetic factors that may contribute to the variability in cancer risks [4, 5].

The *hTERT* gene (MIM 187270) encodes telomerase reverse transcriptase, the catalytic subunit of telomerase, an important protein for maintaining telomere length. Genetic variation within this gene has been shown to affect telomerase activity and telomere length and is thought to underlie an increased risk for cancer [6]. Genetic association studies in breast, ovarian, endometrial, lung, glioma, and pancreatic cancers have identified multiple single nucleotide polymorphisms (SNPs) in the *hTERT* gene that are associated with an increased risk of cancer [7]. Several studies have also identified an association between CRC risk and SNPs within *hTERT* at 5p15.33 [8, 9]. We hypothesized that genetic variation within *hTERT* may act as a genetic modifier of CRC risk for MMR gene mutation carriers.

## RESULTS

A total of 1098 carriers of a germline mutation in a MMR gene (18 in *EPCAM*, 420 in *MLH1*, 481 in *MSH2*, 126 in *MSH6*, and 53 in *PMS2*) from 330 independent families was included in this study. During 46,757 person- years observation, 392 (36%) carriers were diagnosed with CRC at a mean age of 42.2 (standard deviation 11.4) years (Table 1). Of these, 90% of cancer diagnoses were confirmed using pathology reports, medical records, cancer registry reports, and death certificates.

**Table 1:** Characteristics of DNA mismatch repair gene mutation carriers included in the study

	No colorectal cancer (n=706) N (%)	Colorectal cancer (n=392) N (%)	All (n=1098) N (%)
Sex			
Male	273 (38.8)	193 (49.2)	466 (42.5)
Female	433 (61.2)	199 (50.8)	631 (57.5)
Ascertainment			
Population-based	47 (6.7)	41 (10.5)	88 (8.0)
Clinic-based	659 (93.3)	351 (89.5)	1010 (92.0)
Gene mutated			
*EPCAM*	13 (1.8)	5 (1.3)	18 (1.6)
*MLH1*	247 (35.0)	173 (44.1)	420 (38.3)
*MSH2*	317 (45.0)	164 (41.8)	481 (43.8)
*MSH6*	95 (13.4)	32 (7.9)	126 (11.5)
*PMS2*	34 (4.8)	19 (4.9)	53 (4.8)
Age^a^ mean (SD)	43.9 (13.6)	42.2 (11.4)	43.2 (12.8)
Median [range]	42 [9 - 89]	42 [16 - 86]	42 [9 - 89]

a Age at diagnosis for carriers with colorectal cancer; age at diagnosis of other cancer or polypectomy or death or last contact for carriers without colorectal cancer.

**Figure 1:**
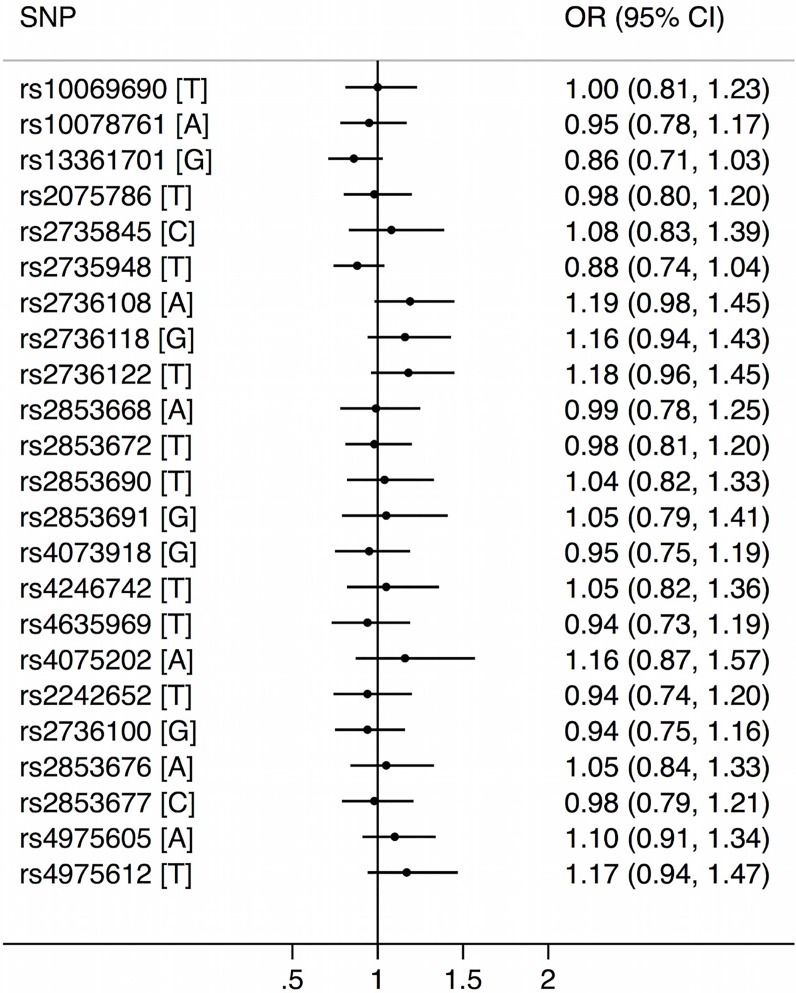
Hazard ratios and corresponding 95% confidence intervals for associations between 23 single nucleotide polymorphisms within the *hTERT* gene and colorectal cancer risk for DNA mismatch repair gene mutation carriers

Of the 23 *hTERT* SNPs that we measured, five SNPs (rs13361701, rs2853691, rs4075202, rs2736100, rs4975612) deviated from Hardy–Weinberg equilibrium in CRC-unaffected carriers (Supplementary Table 1). After adjusting for multiple testing and age and sex of the carriers, there was no evidence of an association between CRC risk and any of these 23 SNPs per allele (Figure 1) or for homozygous or heterozygous carriers of minor allele (Supplementary Table 2). When we stratified by sex of the carriers and the MMR gene mutated, there was also no evidence of any association (detail results not shown). When we censored carriers by age 45 years, we found no evidence of any association (detail results not shown).

We combined the 23 SNPs to examine the effect of haplotypes on CRC risk. Of the 88 haplotypes with frequency greater than 0.002, we estimated the associations between five most common haplotypes and CRC risk. Again, there was no evidence for any association (detail results not shown).

## DISCUSSION

In this study, we examined whether SNPs within the *hTERT* gene act as genetic modifiers of CRC risk for MMR gene mutation carriers. We found no evidence of association between any of the 23 SNPs within the *hTERT* gene and CRC risk either overall or when stratified by sex or specific MMR gene mutation, suggesting that *hTERT* SNPs do not modify the risk of CRC for MMR gene mutation carriers.

A recent study of 930 MMR gene mutation carriers reported no overall difference in genotype frequencies between carriers affected and unaffected with CRC for the rs2075786 SNP in *hTERT* (RR=2.46; 95% CI=0.78–7.82) [10], a finding that is consistent with ours. This study also reported marginally significant evidence of association between the AA genotype of the rs2075786 SNP and cancer risk for carriers diagnosed with Lynch syndrome related-cancer diagnosed before the age of 45 years (RR=2.90; 95% CI=1.02–8.26; p=0.05) but we found no evidence of an association for that SNP and CRC risk before age 45 years.

To date, the search for genetic modifiers of CRC risk for MMR gene mutation carriers has provided inconsistent results. We have shown previously that the 11 SNPs identified from genome-wide association studies (GWAS) of CRC for the general population are not associated with CRC risk for MMR gene mutation carriers [11]. Three studies observed two variants, 8q23.3 (rs16892766) and 11q23.1 (rs3802842), to be associated with increased risk of CRC in MMR gene mutation carriers especially for females only [12, 13] or *MLH1* mutation carriers only [5, 13]. In contrast, another study of 748 mutation carriers did not report this association[14].

Additional SNPs have been identified from CRC GWAS, including the rs2853668 SNP within the TERT- CLPTM1L locus on 5p15.33 [9], but it appears that some of these have no relevance to the risk of CRC for MMR gene mutation carriers. An association between the rs2736100 SNP in *hTERT* and CRC has also been reported in sporadic setting [8] but we found no evidence for an association between this SNP and CRC risk for MMR gene mutation carriers.

Our study has the largest number of MMR gene mutation carriers assessed to date for genetic modifiers of the *hTERT* gene on CRC risk. A possible limitation of our study is its generalizability only to MMR gene mutation carriers with substantial survival, as to be included in the analysis cases had to survive long enough to provide a blood sample for DNA testing. There were 5 SNPs deviated from Hardy–Weinberg equilibrium in CRC- unaffected carriers in our sample. This might be explained by small sample size and non-random sampling of carriers.

In conclusion, our findings suggest that common genetic variants within the *hTERT* gene do not contribute to CRC risk for carriers of MMR gene mutation carriers. More information is important clinically, to provide more accurate risk assessment and risk management. Subsequent studies should focus on testing large cohorts of MMR gene mutation carriers using agnostic genome- wide approaches in order to identify genetic modifiers of risk.

## MATERIALS AND METHODS

### Study recruitment and data collection

The study sample comprised confirmed heterozygote carriers of pathogenic mutations in MMR genes who were participants in the Australasian Colorectal Cancer Family Registry [15]. Between 1997 and 2012, the Australasian Colorectal Cancer Family Registry recruited families via: population-based probands who were recently diagnosed colorectal cancer cases from the Victorian Cancer Registry; or clinic-based probands who were enrolled from multiple-case families referred to family cancer clinics in Melbourne, Adelaide, Perth, Brisbane, Sydney, and Auckland. Written informed consent was obtained from all participants, and the study protocol was approved by the institutional human research ethics committee at each center.

Information on demographics, personal characteristics, personal and family history of cancer, cancer-screening history, and history of polyps, polypectomy, and other surgeries was obtained by questionnaires from all probands and participating relatives. Participants were followed up approximately every five years after baseline to update this information. The present study was based on all available baseline and follow-up data.

### MMR gene mutation testing

Testing for MMR germline mutations was performed for all population-based probands with CRC displaying evidence of impaired MMR function as evidenced by either tumor microsatellite instability (MSI) and/or a lack of MMR protein expression by immunohistochemistry. The youngest-onset colorectal case participants from each clinic-based family were also genotyped, regardless of tumor MSI or MMR protein expression status. Mutation testing for the *MLH1*, *MSH2* and *MSH6* genes was performed by Sanger sequencing or denaturing high performance liquid chromatography, followed by confirmatory DNA sequencing. Large duplication and deletion mutations including those involving *EPCAM*, which lead to *MSH2* methylation, were detected by Multiplex Ligation Dependent Probe Amplification (MLPA). *PMS2* mutation testing involved a modified protocol from Senter et al.[16] where exons 1–5, 9 and 11–15 were amplified in three long range PCRs followed by nested exon specific PCR/sequencing while the remaining exons (6, 7, 8 and 10) were amplified and sequenced direct from genomic DNA. Large-scale deletions in *PMS2* were detected using the P008-A1 MLPA kit (MRC Holland). The relatives of probands with a pathogenic MMR germline mutation, who provided a blood sample, underwent testing for the specific mutation identified in the proband.

### Genotyping of the SNPs

Using data from the International HapMap project (http://www.HapMap.org) and Haploview program (version 3.12) and a minimum r^2^ threshold of 0.8 we identified a set of tagging SNPs to capture the genetic variation in the *hTERT* gene. SNPs previously associated with CRC risk [8, 9, 17] or from the single study of CRC risk in MMR gene mutation carriers [10] were also genotyped. The selected SNPs (Supplementary Table 1) were genotyped using Sequenom's iPLEX Gold. Briefly, PCR and extension primers for these SNPs were designed using the MassARRAY Assay Design 3.0 software (Sequenom, Inc.). PCR amplification and single base extension reactions were performed according to the manufacturer's instructions. Extension product sizes were determined by mass spectrometry using Sequenom's Compact matrix- assisted laser desorption ionization-time of flight mass spectrometer. The resulting mass spectra were converted to genotype data using SpectroTYPER-RT software.

### Statistical analysis

Time-at-risk for each MMR gene mutation carrier started at birth and ended at age at diagnosis of colorectal cancer (n=392), any other cancer (n=141), polypectomy (n=282), death (n=4) or last contact (n=279), whichever occurred first.

Since some carriers were ascertained because they were diagnosed with CRC or they had a strong family history, the identification of MMR gene mutation carriers was not random with respect to the disease status. To adjust for this non-random ascertainment, we used the weighted cohort approach.[18] Age-specific incidence rates of CRC for MMR gene mutation carriers [19] were used to calculate sampling fractions to weight the proportion of affected and unaffected carriers in five-year age stratum so the proportion of affected carriers in each age group equalled that expected for mutation carriers in the population.

Cox proportional hazards regression analysis was used to estimate hazard ratios (HRs) and 95% confidence intervals (CIs) for associations between each of 23 *hTERT* SNPs and CRC risk for MMR gene mutation carriers. We estimated HRs separately for homozygous carriers of the minor allele (2 minor alleles) and heterozygous carriers of the minor allele (1 minor allele) versus non-carriers (0 minor allele); and we estimated HRs per minor allele, i.e. a linear association on the log scale. As a subgroup analysis to investigate the associations between *hTERT* SNPs and early-onset CRC risk, we censored carriers by age of 45 years and conducted regressions.

The proportional hazards assumption was tested by examining the relationship between the scaled Schoenfeld residuals and survival time. To allow for any correlation of risk between family members, the Huber-White robust variance correction was applied by clustering on family membership [20]. To reduce the false discovery rate expected from the large number of associations investigated, the *P* value cut-off for classifying a HR as statistically significant was determined using methods by Benjamini and Hochberg [21]. We put the individual *P*-values in order, from smallest to largest. The smallest *P* value has a rank of *i*=1, then next smallest has *i*=2, etc. Then we compared each individual *P* value to its Benjamini-Hochberg critical value, (*i/m*)*Q*, where *i* is the rank, *m* is the total number of tests, and *Q* is the false discovery rate of 0.05. The largest *P* value that has *P*<(i/m) Q is significant, and *all* of the *P* values smaller than it are also significant. This method controls the expected high false discovery rate and can result in significant gains in power over traditional multiplicity ‘correction’ methods such as the commonly used Bonferroni procedure [22]. All statistical analyses were performed using STATA 13.0 (College Station, TX: StataCorp LP, 2013).

Haplotypes for the 23 SNPs within the *hTERT* gene were estimated using the *haplologit* command in STATA that implements the retrospective profile-likelihood methods of Spinka et al [23]. Logistic regression was used to examine associations between the haplotypes and CRC.

## SUPPLEMENTARY TABLES


