# Supplementary material for: Genetic variants within the hTERT gene and the risk of colorectal cancer in Lynch syndrome
Source: Genes Cancer. 2015 Nov;6(11-12):445–51. doi: 10.18632/genesandcancer.85 (PMC4701223; doi:10.18632/genesandcancer.85)
Supplement: Supplementary file 1 [file ganc-06-445-s001.pdf]

Genetic variants within the *hTERT* gene and the risk of colorectal cancer in Lynch syndrome

Supplementary Table 1. Minor allele frequency of 23 single nucleotide polymorphisms within the *hTERT* gene included in the study

SNP [minor allele]	No colorectal cancer			Colorectal cancer		All	
	Frequency	Observed	P-value[a] for H-W Equilibrium Test	Frequency	Observed	Frequency	Observed
rs10069690 [T]	0.26	372	0.69	0.29	226	0.29	598
rs10078761 [A]	0.34	477	0.88	0.35	272	0.35	749
rs13361701 [G]	0.29	406	<0.001	0.27	207	0.28	613
rs2075786 [T]	0.34	486	0.10	0.36	283	0.35	769
rs2735845 [C]	0.20	288	0.41	0.21	161	0.21	449
rs2735948 [T]	0.39	554	0.28	0.39	303	0.39	857
rs2736108 [A]	0.31	441	0.22	0.32	250	0.31	691
rs2736118 [G]	0.27	386	0.71	0.28	220	0.28	606
rs2736122 [T]	0.27	384	0.31	0.28	220	0.28	604
rs2853668 [A]	0.27	378	0.28	0.27	212	0.27	590
rs2853672 [T]	0.49	669	0.58	0.49	383	0.49	1052
rs2853690 [T]	0.19	256	0.17	0.18	137	0.19	393
rs2853691 [G]	0.18	245	**<0.001**	0.16	128	0.17	373
rs4073918 [G]	0.22	309	0.06	0.23	178	0.22	487
rs4246742 [T]	0.14	196	0.40	0.15	118	0.14	314
rs4635969 [T]	0.19	265	0.15	0.20	154	0.19	419
rs4075202 [A]	0.13	189	**<0.001**	0.16	129	0.14	318
rs2242652 [T]	0.19	272	0.37	0.19	150	0.19	422
rs2736100 [G]	0.49	688	**0.01**	0.48	379	0.49	1067
rs2853676 [A]	0.27	376	0.83	0.27	212	0.27	588
rs2853677 [C]	0.44	614	0.55	0.44	343	0.44	957
rs4975605 [A]	0.46	644	0.99	0.48	407	0.47	1021
rs4975612 [T]	0.22	306	**0.01**	0.22	175	0.22	481

[a] P-value from likelihood ratio test for Hardy–Weinberg equilibrium

Supplementary Table 2. Hazard ratios and corresponding 95% confidence intervals for associations between 23 single nucleotide polymorphisms within the *hTERT* gene and colorectal cancer risk for DNA mismatch repair gene mutation carriers.

	n	N	Hazard ratio	95% confidence interval	P-value
rs10069690					
CC	201	581	1 (Ref)	-	-
TC	156	433	1.00	0.74 – 1.35	0.99
TT	35	82	0.99	0.61 – 1.60	0.627
Per allele [T]	392	1096	1.00	0.81 – 1.23	0.98
rs10078761					
TT	163	462	1 (Ref)	-	-
TA	182	492	0.97	0.73 – 1.29	0.83
AA	45	128	0.89	0.56 – 1.41	0.62
Per allele [A]	390	1082	0.95	0.78 – 1.17	0.63
Rs13361701					
TT	233	628	1 (Ref)	-	-
GT	107	317	0.84	0.62 – 1.15	0.28
GG	50	148	0.74	0.49 – 1.11	0.15
Per allele [G]	390	1093	0.86	0.71 – 1.03	0.10
rs2075786[a]					
CC	159	452	1 (Ref)	-	-
TC	181	518	0.95	0.71 – 1.27	0.74
TT	51	125	0.99	0.65 – 1.50	0.96
Per allele [T]	391	1095	0.98	0.80 – 1.20	0.84
rs2735845					
GG	247	689	1 (Ref)	-	-
CG	127	362	1.00	0.75 – 1.34	0.99
CC	17	43	1.39	0.67 – 2.88	0.37
Per allele [C]	391	1094	1.08	0.83 – 1.39	0.57
rs2735948					
CC	150	414	1 (Ref)	-	-
CT	179	501	0.99	0.75 – 1.30	0.92
TT	62	178	0.70	0.48 – 1.03	0.07
Per allele [T]	392	1093	0.88	0.74 – 1.04	0.12
rs2736108					
GG	182	508	1 (Ref)	-	-
AG	170	487	1.21	0.92 – 1.59	0.18

AA	40	101	1.40	0.86 – 2.28	0.18
Per allele [A]	392	1096	1.19	0.98 – 1.45	0.08
rs2736118					
AA	204	572	1 (Ref)	-	-
AG	154	437	1.16	0.87 – 1.56	0.31
GG	33	84	1.34	0.82 – 2.18	0.23
Per allele [G]	391	1093	1.16	0.94 – 1.43	0.16
rs2736122					
CC	203	571	1 (Ref)	-	-
CT	158	447	1.20	0.90 – 1.61	0.20
TT	31	78	1.35	0.82 – 2.23	0.24
Per allele [T]	392	1096	1.18	0.96 – 1.45	0.12
rs2853668					
CC	209	581	1 (Ref)	-	-
CA	154	442	0.87	0.66 – 1.15	0.34
AA	29	74	1.19	0.70 – 2.04	0.52
Per allele [A]	393	1097	0.99	0.78 – 1.25	0.93
rs2853672					
GG	108	293	1 (Ref)	-	-
GT	181	518	0.76	0.53 – 1.08	0.16
TT	101	267	0.97	0.68 – 1.40	0.88
Per allele [T]	390	1078	0.98	0.81 – 1.20	0.86
rs2853690					
CC	258	694	1 (Ref)	-	-
TC	119	337	1.03	0.79 – 1.35	0.81
TT	9	28	1.13	0.47 – 2.72	0.79
Per allele [T]	386	1059	1.04	0.82 – 1.33	0.74
rs2853691					
AA	262	708	1 (Ref)	-	-
AG	128	369	1.05	0.79 – 1.41	0.69
GG	0	2	-	-	-
Per allele [G]	390	1079	1.05	0.79 – 1.41	0.72
rs4073918					
AA	229	642	1 (Ref)	-	-
GA	142	399	0.96	0.72 – 1.29	0.80
GG	18	44	0.84	0.49 – 1.46	0.54
Per allele [G]	389	1085	0.95	0.75 – 1.19	0.65
rs4246742					

AA	282	801	1 (Ref)	-	-
AT	100	274	1.06	0.78 – 1.44	0.71
TT	9	20	1.06	0.56 – 2.01	0.86
Per allele [T]	391	1095	1.05	0.82 – 1.36	0.69
rs4635969					
CC	252	719	1 (Ref)	-	-
TC	126	329	1.01	0.74 – 1.38	0.95
TT	14	45	0.70	0.35 – 1.42	0.33
Per allele [T]	392	1093	0.94	0.73 – 1.19	0.59
rs4075202					
CC	263	782	1 (Ref)	-	-
CA	129	313	1.20	0.88 – 1.63	0.25
AA	0	2	-	-	-
Per allele	392	1096	1.16	0.87 – 1.57	0.31
rs2242652					
CC	260	722	1 (Ref)	-	-
CT	114	326	1.00	0.73 – 1.37	0.99
TT	18	48	0.76	0.41 – 1.41	0.38
Per allele [T]	392	1096	0.94	0.74 – 1.20	0.63
rs2736100					
TT	100	266	1 (Ref)	-	-
GT	205	588	0.75	0.54 – 1.03	0.08
GG	87	239	0.90	0.60 – 1.35	0.62
Per allele [G]	392	1093	0.94	0.75 – 1.16	0.55
rs2853676					
GG	215	593	1 (Ref)	-	-
GA	142	419	0.91	0.68 – 1.22	0.52
AA	35	84	1.38	0.83 – 2.31	0.22
Per allele [A]	392	1096	1.05	0.84 – 1.33	0.65
rs2853677					
TT	175	353	1 (Ref)	-	-
TC	133	528	0.70	0.52 – 0.94	**0.02**
CC	84	214	1.08	0.74 – 1.57	0.70
Per allele [C]	392	1095	0.98	0.79 – 1.21	0.87
rs4975605					
CC	96	304	1 (Ref)	-	-
CA	215	565	1.39	1.01 – 1.91	0.04
AA	81	227	1.18	0.78 – 1.80	0.44

Per allele [A]	392	1096	1.10	0.91 – 1.34	0.32
rs4975612					
GG	241	684	1 (Ref)	-	-
GT	127	342	1.12	0.83 – 1.53	0.44
TT	24	69	1.45	0.83 – 2.54	0.19
Per allele [T]	392	1095	1.17	0.94 – 1.47	0.17

[a] reported in Bellido et al study of 255 and 675 MMR gene mutation carriers from Spain and the Netherlands, respectively [13]
